# Dynamic self-navigated 3D whole-heart radial coronary MRA with retrospective acquisition window selection

**DOI:** 10.1186/1532-429X-16-S1-O18

**Published:** 2014-01-16

**Authors:** Simone Coppo, Davide Piccini, Jerome Chaptinel, Gabriele Bonanno, Matthias Stuber

**Affiliations:** 1 Radiology, University Hospital of Lausanne (CHUV) and University of Lausanne (UNIL), Lausanne, Switzerland; 2 Center for Biomedical Imaging (CIBM), Lausanne, Switzerland; 3 Advanced Clinical Imaging Technology, Siemens Healthcare IM BM PI, Lausanne, Switzerland

## Background

Navigator gated coronary MRA is highly time inefficient as the data collection duty cycle (DC) is only 2% [1]. Here, we report a new, dynamic self-navigated coronary MRA technique with isotropic spatial resolution that a) further improves DC by continuously acquiring image data without ECG triggering and that b) enables retrospective, flexible, and individual selection of the acquisition window in the cardiac cycle.

## Methods

**Figure 1:**
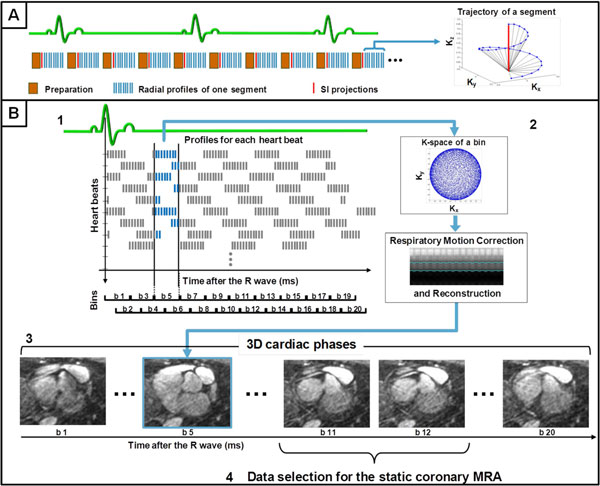
**(A) Schematic representation of the continuous acquisition, on the left, with an example of a 3D trajectory of one k-space segment (top hemisphere) on the right**. The SI readout used for respiratory self-navigation is highlighted in red. Each data segment is rotated with the golden angle with respect to the previous one. (B) Example of the binning of the readouts for one cardiac phase: 1) Data originating from a specific time interval (100 ms width) after the R-wave are grouped together (blue); 2) respiratory self navigation is then applied and a 3D cine frame reconstructed. 3) This is repeated multiple times each 50 ms for full coverage of the cardiac cycle. 4) The period of most quiescent coronary motion was identified and data coming from this time interval are used to reconstruct the final, static coronary MRA dataset.

Dynamic self-navigated whole-heart coronary MRA was implemented on a 1.5T clinical scanner (Aera, Siemens, Germany) and tested in 5 healthy adult volunteers. Imaging was performed with segmented bSSFP: TR/TE = 3.1/1.56 ms, FOV = (220 mm)^3, isotropic voxel size = (1.15 mm)^3, matrix size = 192^3, radiofrequency excitation angle = 90°. Data were acquired continuously during 14 min, using a 3D radial trajectory with a spiral phyllotaxis pattern [2] without ECG triggering (Figure 1A). Each segment, preceded by fat saturation, was rotated by the golden angle (137.51°) relative to its predecessor. Sampling uniformity was thus obtained automatically. After scan completion, radial profiles were regrouped in temporal bins (Figure 1B) leading to a reconstructed 3D dataset every 50 ms covering the entire cardiac cycle. Self-navigation for respiratory motion compensation was then performed [3]. From the thus obtained 3D cine datasets, the onset and duration of the period of most quiescent coronary motion was identified, and profiles acquired during this time window were retrospectively selected to reconstruct 3D whole heart coronary MRA.

## Results

**Figure 2:**
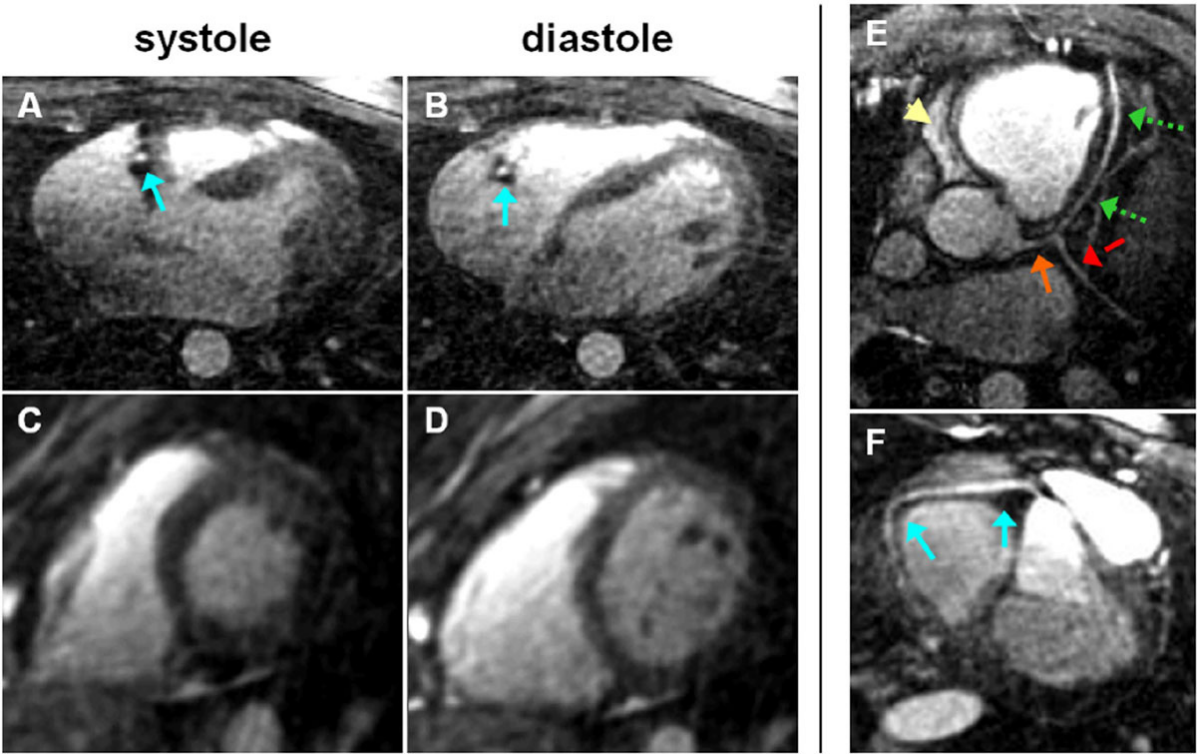
**Systolic (A, C) and diastolic (B, D) cine frames extracted from the non-ECG triggered self-navigated 3D acquisition in a healthy adult volunteer (axial view in A, B and short axis view in C, D)**. In E & F, coronary MRA from two different subjects and reconstructed from a retrospectively selected, diastolic acquisition window, are displayed. In E, the left main (orange arrow), the left anterior descending (green dotted arrows) and the left circumflex (red dashed arrow) coronaries are visualized, while a right coronary artery (blue solid arrows) is shown in (F). Residual fat signal is still visible in the image (yellow arrowhead).

Dynamic coronary MRA were successfully obtained in all volunteers with a DC of 16% (as compared to 4% for self-navigation alone [2] and to 2% for conventional navigator techniques). In our cohort, the optimal acquisition window for retrospective coronary MRA reconstruction was always found in diastole. This is visualized in Figure 2E &2F, where diastolic left and right coronary arterial systems extracted from individual time intervals are displayed. Note the high blood-muscle contrast despite the absence of a T2Prep. Residual fat signal (arrowhead) is still observed.

## Conclusions

Non-ECG triggered dynamic self-navigated 3D whole-heart radial coronary MRI with isotropic spatial resolution was successfully implemented in vivo for the first time. It improves DC and enables a retrospective and flexible selection of the acquisition window for coronary MRA data reconstruction. While improvements in signal-to-noise ratio and fat saturation are necessary, this provides a first step toward a coronary MRA approach for which the time frame with the best depiction of a selected coronary artery segment can freely be chosen.

## Funding

This work was in part supported by the Swiss National Science Foundation grant #320030-143923.
